# Efficacy and safety of a modular multi-modal exercise program in prostate cancer patients with bone metastases: a randomized controlled trial

**DOI:** 10.1186/1471-2407-11-517

**Published:** 2011-12-13

**Authors:** Daniel A Galvão, Dennis R Taaffe, Prue Cormie, Nigel Spry, Suzanne K Chambers, Carolyn Peddle-McIntyre, Michael Baker, James Denham, David Joseph, Geoff Groom, Robert U Newton

**Affiliations:** 1 Edith Cowan University Health and Wellness Institute and School of Exercise and Health Sciences, Edith Cowan University, Joondalup, Australia; 2 School of Environmental and Life Sciences, University of Newcastle, Ourimbah, Australia; 3 Department of Radiation Oncology, Sir Charles Gairdner Hospital, Nedlands, Australia; 4 Faculty of Medicine, University of Western Australia, Nedlands, Australia; 5 Griffith Health Institute, Griffith University, Gold Coast, Australia; 6 Viertel Centre for Research in Cancer Control, Cancer Council Queensland, Brisbane, Australia; 7 School of Medicine and Public Health, University of Newcastle, Newcastle, Australia; 8 Newcastle Mater Hospital, Newcastle, Australia; 9 Perth Radiological Clinic, Joondalup Health Campus, Joondalup, Australia

## Abstract

**Background:**

The presence of bone metastases has excluded participation of prostate cancer patients in exercise intervention studies to date and is also a relative contraindication to supervised exercise in the community setting because of concerns of fragility fracture. However, this group of patients often have developed significant muscle atrophy and functional impairments from prior and continuing androgen deprivation that is exacerbated by subsequent and more intensive interventions such as chemotherapy. The aim of this study is to determine the efficacy and safety of a modular multi-modal exercise program in prostate cancer patients with bone metastases.

**Methods/Design:**

Multi-site randomized controlled trial in Western Australia and New South Wales to examine the efficacy and safety of a modular multi-modal physical exercise program in 90 prostate cancer survivors with bone metastases. Participants will be randomized to (1) modular multi-modal exercise intervention group or (2) usual medical care group. The modular multi-modal exercise group will receive a 3-month supervised exercise program based on bone lesion location/extent. Measurements for primary and secondary endpoints will take place at baseline, 3 months (end of the intervention) and 6 months follow-up.

**Discussion:**

Delaying or preventing skeletal complication and improving physical function for men with bone metastases would provide clinically meaningful benefits to patients. However, exercise programs must be designed and executed with careful consideration of the skeletal complications associated with bone metastatic disease and cumulative toxicities from androgen deprivation such as osteoporosis and increased risk of fractures. The results from this study will form the basis for the development of a specific exercise prescription in this patient group in order to alleviate disease burden, counteract the adverse treatment related side-effects and enhance quality of life.

**Trial Registration:**

ACTRN: ACTRN12611001158954

## Background

Metastases to bone occurs in approximately 80% of men with advanced prostate cancer [1] and the majority of these patients are at risk of developing pathological fractures, hypercalcemia, bone marrow suppression and nerve compressions or spinal cord compressions that result in significant morbidity, limited function and decreased quality of life [2-4]. The clinical course of metastatic bone disease in prostate cancer survivors is relatively long, with a 5-year survival rate of approximately 30% [5]. Prostate cancer causes predominately sclerotic lesions and commonly metastasize to the pelvis and axial skeleton [6]. Therefore, patients with bone metastases experience considerable morbidity resulting from skeletal complications and fatigue secondary to chemotherapy for those with castrate-resistance prostate cancer [3,7]. Delaying or preventing skeletal complication, improving physical function and increasing levels of physical activity in prostate cancer patients with bone metastases can provide clinically meaningful benefits to patients.

Long-term androgen deprivation therapy (ADT) remains the first-line treatment for advanced metastatic prostate cancer, however it acts by inducing severe hypogonadism causing a number of cumulative adverse effects [8,9]. Some of these physical side effects include reduction of bone and muscle mass, increased fat mass and loss of neuromuscular strength [8,10]. We have shown that a 9-month exposure to ADT led to significant reductions in hip (1.5%), spine (3.9%), whole body (2.4%) and upper limb (1.3%) bone mineral density (BMD) [11]. We have also reported that men on ADT had significantly reduced upper- and lower-body muscle strength and impaired overall functional performance and balance compared to healthy aged-matched controls [12]. Notably, ADT has been associated with increased fracture risks and more importantly this risk increases with the number of doses of gonadotropin-releasing hormone agonist administrated [8]. These observations raise a major concern for men receiving long-term ADT for advanced prostate cancer, with reduced muscle strength placing prostate cancer patients at higher risk of falling particularly when combined with ADT induced reductions in bone strength, all of the sequelae of falls and fracture. This is a major concern as fractures cause significant morbidity and mortality in men [13] and has been correlated with decreased survival in prostate cancer patients with bone metastases [14].

Apart from previous ADT, men with bone metastases and castrate-resistant prostate cancer would commonly receive chemotherapy given the results of initial phase III trials indicating modest survival benefit for those with symptomatic disease [15,16]. A number of well-established adverse effects including nausea and severe fatigue are associated with chemotherapy [15]. Therefore, prostate cancer survivors with bone metastases suffer not only from the treatment-related side effects common to survivors with localised disease, but also from significant physical [17] and psychological [18,19] issues associated with bone metastatic disease and its treatment.

Clinical trials investigating the efficacy of exercise in prostate cancer survivors including our recent randomized controlled trial excluded those who had bone metastases [20-24], or bone lesions deemed 'unstable'[25] due to the potential increased risk of skeletal fractures. Nevertheless, recent guidelines on Exercise for Cancer Survivors by the American College of Sports Medicine [26] suggest that cancer survivors including those with bone metastases should "avoid inactivity" given the potential benefits of physical activity even for this group of cancer patients with advanced disease. However, it remains to be determined if exercise can be tolerated by patients with bone metastases given the absence of clinical data on exercise feasibility and efficacy in patients with bone metastases. This situation is highly detrimental since patients with bone metastases are reducing their physical activity levels for fear of bone fracture and clinicians are reluctant to refer patients with bone metastases for physical exercise programs. Such a strategy can only result in greater fatigue, reduced function and further declining muscle and skeletal integrity, greater risk of other chronic disease and reduced quality of life [3,4]. This project is unique as it tests the implementation of a modular multi-modal exercise program (M3EP) taking into consideration location/extent of bone metastases lesions as a strategy to maintain or enhance physical function in this group of patients with advanced prostate cancer. Further, the outcomes from this trial may provide novel data that could be translated to other cancer patients with bone metastases. This is the first trial to our knowledge which is specifically designed to address the potential beneficial effects of exercise in cancer patients with bone metastases.

## Methods/Design

This is a two-armed prospective randomized controlled trial that will examine the efficacy and safety of a modular multi-modal physical exercise program in prostate cancer survivors with bone metastases. The trial will be a multisite study with clinical sites in Western Australia and New South Wales. Participants randomly assigned to the M3EP exercise intervention group will receive a 3-month supervised exercise intervention program (based on bone lesion location/extent) (Table 1). Participants in the usual medical care group will be asked not to change their baseline levels of physical activity and will be offered the same exercise program after the 3-month control period if the intervention is deemed to be feasible and efficacious.

**Table 1:** Summary of study design

Months	0			3			6
**Exercise **(M3EP)		Intervention (n = 45)			Follow-up from 3-6 month		

**Usual Medical Care**		Usual Care (n = 45)			**Usual Medical Care **to receive same intervention (M3EP) from 3-6 month		

### Recruitment

Subjects will be recruited by invitation of their specialist (radiation oncologist/urologists). Those entering the study will undertake a series of familiarisation sessions and baseline measurements prior to randomisation.

### Randomisation

Patients will be randomly allocated in a ratio of 1:1 to the two study arms for the experimental M3EP exercise intervention or usual medical care groups, subject to maintaining approximate balance regarding stratification for current chemotherapy (yes/no). A research methods consultant with no patient contact will be responsible for randomisation. The exercise physiologists and other researchers conducting the study measures will be blinded to a given participant's group allocation. The exercise intervention will be provided by exercise physiologists not in the research team or performing the tests.

### Subjects

Ninety men (45 subjects per arm) with prostate cancer and established bone metastases with no regular exercise (undertaking structured aerobic or resistance training two or more times per week) within the past 3 months will be recruited through invitation by their attending specialist in Perth, Western Australia, and the Central Coast region of New South Wales. All participants will require physician consent. Exclusion criteria will include acute illness, significant bone pain, musculoskeletal or cardiovascular or neurological disorders that could inhibit or put them at risk from exercising. The protocol has been approved (ID: 7699 GALVÃO) by the Edith Cowan University Human Research Ethics Committee and all participants will provide written informed consent.

### Calculation of sample size

Data from our research team in prostate cancer patients indicates that the standard deviation (SD) for change in our primary outcome which is the physical function subscale of the Medical Outcomes Study Short-Form 36 (SF-36) equates to ~12 points following a 3-month intervention. Given the health status of men in the proposed study we anticipate that our 3-month exercise regimen will result in an increase of ~5 points in the physical function subscale of the SF-36 whereas the usual medical care group will result in an overall loss of ~3 points over 3 months. Therefore, we anticipate a difference between the exercise and usual medical care groups of ~8 points in change of the physical function subscale from baseline to 3 months. A priori, 36 subjects per group will be required to achieve 80% power at an alpha level of 0.05 (two-tailed), and to demonstrate a difference between groups at the end of the 3-month intervention. Therefore, to adequately ensure that we have sufficient subject numbers at the end of the intervention (accounting for a drop-out rate of ~20%), 90 subjects will be randomized in a ratio of 1:1 to exercise and usual medical care groups, respectively.

### Measurements

Measurements for primary and secondary endpoints will take place at baseline, 3 months (end of the intervention) and 6 months follow-up.

#### Primary study endpoints

##### Physical function

The physical function subscale of the Medical Outcomes Study Short-Form 36 (SF-36) questionnaire will be used as an indicator of patient rated physical functioning.

#### Secondary study endpoints

##### Objective measures of physical function

A battery of tests will be used to assess functional performance [22,23,27]. Tests will be performed in triplicate (except for the 400-m walk which will be performed once) with sufficient recovery time between trials. The best performance on each test will be used in the analyses. The tests will be: 1) timed up and go, 2) 6-meter walk, usual and fast pace, and 3) 400-m walk. Participants with proximal femur bone lesion will be excluded from the 400-m walk test. Performance in each test will be timed electronically using a Kinematic Measurement System (Fitness Technology, SA, Australia).

##### Muscle strength

Dynamic muscular strength of the upper and lower body will be assessed using the one repetition maximum (1RM) method [28]. The 1RM is the maximal weight an individual can move through a full range of motion without change in body position other than that dictated by the specific exercise motion. Participants will perform 1RM tests for the knee extension and chest press exercises using a standard 1RM protocol [23]. Participants with proximal femur bone lesion will be excluded from leg extension 1-RM. Participants with axial skeleton (thoracic/ribs) bone lesion will be excluded from chest press 1-RM. These exercises were selected as they do not involve compression of the spine or excessive load in the pelvic area.

##### Balance and risk of falling

A Neurocom Smart Balancemaster (Neurocom, OR, USA) will be used to assess static and dynamic balance. This device measures ground reaction force to track whole body centre of pressure and a tilting visual field and support platform to separate the visual, somatosensory and vestibular balance sense of the patient [23]. Falls self-efficacy will be determined using the Activities-Specific Balance Confidence scale [29]. During the course of the intervention, all participants will record any falls that take place and submit monthly fall records to the investigators.

##### Safety of the exercise program

The safety of the exercise program will be assessed by recording the incidence and severity of any adverse events and skeletal complications throughout the intervention. Skeletal complications include pain at known bone metastases sites and pathological skeletal fractures [17]. Bone pain will be monitored according to the Common Terminology Criteria of the National Cancer Institute: grade 1 mild, not interfering with function; grade 2 moderate pain interfering with function but not interfering with the activities of daily life; and grade 3 severe pain, severely interfering with the activities of daily living. In the presence of bone pain, patients will cease the exercise program and undergo standard clinical evaluation, including plain x-rays and other more specialised imaging, as deemed appropriate.

##### Body composition

Regional and whole body lean mass (including appendicular skeletal muscle mass) and fat mass will be derived from a whole body dual-energy X-ray absorptiometry scan (Hologic Discovery A, Waltham, MA). Measurement of trunk adiposity is an important indicator of chronic disease risk, and will be assessed from trunk fat mass obtained from the whole body scan and the ratios of trunk fat to limb fat, and trunk fat to total fat.

##### Muscle density and cross-sectional area

Peripheral Quantitative Computed Tomography (XCT3000, Stratec, Pforzheim, Germany) will be used to measure muscle density (an indicator of fat infiltration within the muscle and hence muscle quality) and muscle cross-sectional areas of the upper and lower limbs [30].

##### Health-related quality of life, bone pain and the late life - function index

Health-related quality of life outcomes on general health, pain, vitality, social functioning, emotional role, and mental health will be measured using the SF-36 [31]. The FACIT-Bone Pain questionnaire will be used to assess the nature, severity and impact of bone pain [32]. The Late Life - Function Index (LL-FI) will be used to assess patient-reported physical functioning [33].

##### Anxiety and depression

The Brief Symptom Inventory-18 will provide a global measure of current psychological distress with subscale scores for anxiety, depression, and somatisation [34].

##### Cancer specific distress

The Impact of Events Scale (IES) and the Memorial Anxiety Scale for Prostate Cancer (MAX-PC) will be used to measure cancer specific distress [35,36]. The IES has 15 items and contains two subscales: Intrusion and Avoidance [36]. Intrusion can also be used as a proxy measure for rumination about cancer. The MAX-PC consists of 18 items and assesses cancer specific distress across three domains: Prostate Cancer Anxiety; Prostate Specific Antigen (PSA) Anxiety; and Fear of Recurrence [35].

##### Fatigue

Fatigue will be assessed using the Functional Assessment of Chronic Illness Therapy-Fatigue (FACIT-F) questionnaire. The FACIT-F is a 13-item scale commonly used to assess fatigue in cancer patients [37] as well as cancer patients receiving exercise interventions [38].

##### Sleep quality

Items from the Pittsburgh Sleep Quality Index (PSQI) will be used to measure sleep quality [39]. The PSQI is used to asses quality of sleep over a 1-month interval, and has been shown to be reliable and sensitive to change [40].

##### Physical activity motivation

The Theory of Planned Behaviour (TPB) is the most widely utilised behavioural framework when examining physical activity motivation in cancer survivors [41]. Therefore, physical activity motivation will be assessed in accordance with the TPB. TPB constructs (affective and instrumental attitude, injunctive and descriptive norm, self-efficacy, perceived behavioural control, intention, and planning) will be assessed in accordance with established guidelines [41].

##### Physical activity level

Self report physical activity level will be assessed by the Godin Leisure-Time Questionnaire. ActiGraph activity monitors (triaxial accelerometer) will be used to objectively assess physical activity levels over a 7-day period [42]. A 6-item sedentary questionnaire will be used to assess the level of sedentary behaviour.

##### Tolerance of the program

Tolerance of the exercise program will be evaluated by recording participants' ratings of perceived exertion on a Borg scale after every exercise session. Additionally, a custom designed survey examining exercise tolerance using a 7-point Likert scale will be administered prior to the first exercise session each week. The number of participants completing the 3-month program as well as the number of sessions attended will be recorded.

#### Other monitoring measures

##### Prostate specific antigen (PSA)

PSA will be assessed at baseline, 3 months (end of the intervention) and 6 months follow-up by an accredited National Association of Testing Authorities laboratory (Pathwest Diagnostics, Perth, Western Australia).

#### Exercise intervention

The modular multi-modal physical exercise intervention program (M3EP) will comprise resistance, aerobic and flexibility exercises undertaken 3 times per week in an exercise clinic setting supervised by an exercise physiologist. Exercise training sessions will take approximately 60 min (this includes the warm-up and cool-down periods) and will be conducted in the Exercise Clinics at Edith Cowan University in Perth, and at the University of Newcastle's Central Coast campus in Ourimbah. The programme will include 6 exercises that target the major trunk, upper and lower body muscle groups, which we have used in a number of previous studies [21-23,27,43-46] including men on ADT. There will be a modular component for the resistance exercise prescription based on location/extent of bone metastases (Table 2). To ensure the progressive nature of the training program, subjects will be encouraged to work past the specific repetition maximums (RMs) prescribed. The resistance will be increased by 5-10% increment for the next set/training session if subjects are able to perform more repetitions than the RMs specified during a set. Moderate intensity and volume of resistance exercise will range from 10 to 12-RM (e.g. the maximal weight that can be lifted 10 to 12 times) using 3 sets per exercise. This program differs considerably from those commonly prescribed for survivors with localised prostate cancer. Specifically, the M3EP is designed to minimise compressive and shear loads on affected skeletal sites to account for the reduced load bearing capabilities of bone due to metastatic disease in specific regions. Such loading is considerably below the forces exerted on the skeleton during tasks of daily living such as descending stairs or stepping down from a height and will be similar to that of walking. All exercises will be performed at a set cadence of 2 s for both eccentric and concentric phases further minimizing peak forces transmitted to the skeleton. Aerobic exercise component of the M3EP will also be based on location/extent of bone metastases with those with pelvis, axial skeleton (lumbar), proximal femur and all regions bone metastatic disease undergoing non-weight bearing activities (Table 2). The aerobic component will include 20-30 min of cardiovascular exercise using various modes such as walking on a treadmill, cycling or rowing a stationary ergometer, or exercising on a cross training machine based on disease extent (Table 2). Target intensity will be 60%-85% estimated maximum heart rate (220 - age) with individual heart rate monitors (Polar Electro Oy, Finland) provided for each participant. The flexibility component of the M3EP will involve static stretching of all the joint ranges of motion considered important for function. Those with axial skeleton and all regions bone metastases will be excluded from undertaking spine flexion/extension/rotation (Table 2). The protocol will involve 2-4 sets per muscle group at 30-60 s per set as previously proposed [47]. All M3EP sessions will be conducted in small groups of up to 8 participants under direct supervision to ensure correct technique and minimal risk of injury. Each session will commence with a 10-minute warm-up comprising low-level aerobic activities such as treadmill walking and stationary cycling as determined by bone metastases site, as well as stretching and conclude with a 5-minute cool-down period of stretching activities. The M3EP will be designed to provide adequate stimulus to the cardiorespiratory, skeletal and neuromuscular systems while maximizing compliance and retention. All participants will be asked to maintain customary physical activity and dietary patterns over the intervention period (apart from the programmed exercise). Dietary intake will be assessed at baseline, 3 and 6 months using 3-day record. During the course of the study, participants will be required to maintain an activity log and record their recreational physical activities.

**Table 2:** Modular multi-modal physical exercise program (M3EP) for prostate cancer with bone metastases

Metastases site	Exercise mode					
	
	*Resistance*			*Aerobic*		*Flexibility*
	**Upper**	**Trunk**	**Lower**	**WB**	**NWB**	**Static **

Pelvis	√	√	√**		√	√

Axial Skeleton (lumbar)	√		√		√	√***

Axial Skeleton (thoracic/ribs)	√*		√	√	√	√***

Proximal Femur	√	√	√**		√	√

All regions	√*		√**		√	√***

√ = Target exercise region; * = exclusion of shoulder flexion/extension/abduction/adduction - inclusion of elbow flexion/extension; ** = exclusion of hip extension/flexion - inclusion of knee extension/flexion; *WB *weight bearing (e.g. walking); *NWB *non-weight bearing (e.g. cycling); *** = exclusion of spine/flexion/extension/rotation.

### Statistical analysis

Data will be analysed using the SPSS statistical software package and an intention-to-treat approach will be applied. Analyses will include standard descriptive statistics, Student's t tests, correlation and regression, and two-way (group × time) repeated measures ANOVA (or ANCOVA as appropriate) to examine differences between groups over time. All tests will be two-tailed and an alpha level of 0.05 will be applied as the criterion for statistical significance.

## Discussion

Prostate cancer patients with bone metastases experience considerable morbidity resulting from skeletal complications and often fatigue secondary to chemotherapy [3,7]. Delaying or preventing skeletal complication and improving physical function for men with bone metastases would provide clinically meaningful benefits to patients. However, exercise programs must be designed and executed with careful consideration of the skeletal complications associated with bone metastatic disease and cumulative toxicities from androgen deprivation such as osteoporosis and increased risk of fractures. Currently, such patients are unable to follow existing exercise guidelines established for patients with localised prostate cancer given the absence of exercise-related data for this population. Consequently, the results from this study will form the basis for the development of a specific exercise prescription in this patient group in order to alleviate disease burden, counteract the adverse treatment related side-effects and enhance quality of life. This project is unique as it will be the first study examining if a modular multi-modal targeted exercise program incorporating resistance, aerobic and flexibility exercise is safe and well tolerated by prostate cancer patients with bone metastases. In terms of advancement of prostate cancer care, we expect dissemination of the knowledge gained from this project to reduce fracture risk, improve physical and functional ability, quality of life and ultimately survival rates in this population. Lastly, the proposed study will provide strong, innovative information that has the potential to directly influence current clinical recommendations for other advanced cancer patients with bone metastases. Although the intervention in this study will be highly supervised and targeted to patient's specific needs, given the nature of the modular prescription approach, it has the potential to be performed and implemented in different centres and at the community level thereby reaching a significant number of patients.

## Abbreviations

ADT: Androgen deprivation therapy; IES: Impact of events scale; MAX-PC: Memorial anxiety scale for prostate cancer; M3EP: Modular multi-modal exercise program; PSA: Prostate specific antigen; SF-36: Medical outcomes study short-form 36; FACIT-F: Functional assessment of chronic illness therapy-fatigue; TPB: Theory of planned behaviour; LL-FI: The late life - function index; PSQI: Pittsburgh sleep quality index.

## Competing interests

The authors declare that they have no competing interests.

## Authors' contributions

DAG, PC, NS, DRT, and RUN developed the study concept and protocols and initiated the project. SKC, CPM, MB, JD, GG and DJ assisted in further development of the protocol. DAG, PC, DRT and RUN drafted the manuscript. NS, JD, and DJ will provide access to patients. DAG, PC, DRT, CPM, MB and RUN will implement the protocol and oversee collection of the data. All authors contributed and approved the final manuscript.

## Pre-publication history

The pre-publication history for this paper can be accessed here:

http://www.biomedcentral.com/1471-2407/11/517/prepub
